# Initial health assessments for newly arrived migrants, refugees, and asylum seekers

**DOI:** 10.1136/bmj-2021-068821

**Published:** 2022-04-28

**Authors:** Felicity Knights, Shazia Munir, Haja Ahmed, Sally Hargreaves

**Affiliations:** 1 The Migrant Health Research Group, Institute for Infection and Immunity, St George’s, University of London, London, UK; 2 Refugee and Asylum Seeker Services, Health Inclusion Team, Guy’s & St Thomas’ NHS Foundation Trust, London, UK; 3 London, UK

**What you need to know**

- Consider screening for communicable diseases (including active and latent TB, hepatitis B/C, HIV, and parasitic infections) dependent on country of origin, and offer catch up vaccinations for all newly arrived children, adolescents, and adults to align with the host nation’s schedule
- Non-communicable diseases may be undiagnosed or poorly controlled; maintain and review medication supplies
- Consider nutritional deficiency, oral health, pregnancy, contraception, mental health, and traumatic experiences
- Show kindness and empathy during all encounters, as interaction with healthcare workers can markedly influence migrants’ lives in new countries. Take a holistic, person-centred approach, and signpost patients to services, voluntary support, and translated health information

Migrant, refugee, and asylum seeker populations in Europe have increased in recent years, including in response to the current conflicts in Afghanistan, Syria, and—more recently—Ukraine.^1^ ^2^ ^3^ Countries neighbouring those in crises, and transit countries, are most affected, but so are many other host nations across the world.^1^ ^2^ ^3^

People fleeing conflict or humanitarian crisis, undocumented migrants, refugees, asylum seekers, and people who have been trafficked, may be more vulnerable than other migrants.^4^ Health service delivery to these groups can be complex and has implications for health systems and front line clinicians tasked with meeting the needs of these diverse populations.

This article outlines how primary care services and multidisciplinary teams can meet the initial healthcare needs of newly arrived migrants. We include some specific new guidance on health provision for newly displaced populations from Ukraine; however, the main focus is on refugees and asylum seekers from Afghanistan who have arrived in the UK in large numbers over the past year or so, and for whom UK GPs have requested specific guidance. That said, the advice presented is broadly applicable to all countries hosting migrant groups from any country.

Evidence regarding best practice for migrants as a patient group is limited. This article draws primarily on the available specialist guidance and the authors’ clinical and professional experience.

## What is an effective consultation?

In the UK, everyone has the right to register and consult with a GP (boxes 1 and 2). Entitlements vary between countries and migrant groups. Where required, provide support in registration, navigation, and attendance of appointments, remembering that digital registration and triage systems introduced during the covid-19 pandemic may pose challenges. All services have a role in signposting and supporting access to a full and holistic assessment, regardless of where patients initially present.

Box 1 **UK NHS entitlements**

**Primary care**

- Everyone living in the UK is entitled to register and consult with a GP, free of charge
- Proof of address, identification, and immigration status are not required for GP registration.

**Secondary care**

- NHS services that are free regardless of immigration status include:
  - accident and emergency services
  - NHS 111 advice service
  - diagnosis and treatment of communicable diseases
  - diagnosis and treatment of sexually transmitted infections
  - testing and treatment of covid-19
  - family planning services
  - palliative care services provided by charity or private provider
  - treatment for conditions caused by torture, female genital mutilation, domestic or sexual violence.
- Groups who are eligible for free secondary care are:
  - refugees, asylum seekers, and their dependants
  - refused asylum seekers living in Northern Ireland, Scotland, and Wales
  - refused asylum seekers living in England, who receive Section 95 or Section 4^2^ support
  - children looked after by the local authority
  - immigration detainees
  - people who have been subject to modern slavery and human trafficking
  - people treated under the Mental Health act.
- For groups that are chargeable for secondary care, such as undocumented migrants, *urgent* or *immediately necessary* care must be given, regardless of ability to pay. Maternity services are always *immediately necessary*

**Maternity care**

Maternity care is free for refugees and asylum seekers. For groups that may be chargeable, maternity services are always immediately necessary.

Refugees, asylum seekers, and other migrants may be able to access free vitamins (folic acid, vitamins C and D) through the Healthy Start Scheme.

Maternity prescription exemption forms should be provided.

**Help with prescription charges, dental, and optical care**

People with low income, including refugees, asylum seekers, refused asylum seekers, and undocumented migrants, can apply to the NHS low income scheme for help with medical costs, including prescription charges, dental treatment, and sight tests. Individuals must fill in an HC1 form and will receive an HC2 certificate if eligible. A maternity exemption form entitles pregnant patients to free dental treatment. Further details are available in the ‘Patient resources’ box, below.

Box 2 **Refugees and asylum seekers from Afghanistan in the UK**

- Resettled refugees—The UK government has established the Afghan Relocations and Assistance Policy to resettle those who supported British efforts in Afghanistan.^5^ In addition, the Afghan Citizens Resettlement Scheme has committed to welcome 5000 refugees in the first year and 20 000 in the coming years.^6^ Clinical guidance is available specifically for Afghans arriving via these schemes^5^
- Asylum seekers—1974 applications for asylum from Afghan nationals were filed in the year ending September 2021.^7^ This number is likely to rise and does not include unknown numbers of undocumented Afghan migrants who have not submitted an asylum claim. Unlike resettled refugees, these individuals do not receive the same benefits, are not able to work, and live with the uncertainty of the outcome of their asylum claim
- Arrivals from Afghanistan have typically been housed in contingency and temporary accommodation such as hotels. In many parts of the country, individuals have remained in these settings for many months awaiting more permanent accommodation

The UK Health Security Agency (UKHSA) recommends inquiring about circumstances before migration, the journey, and current circumstances (including discrimination) within a person centred assessment of any presenting migrant patient.^8^

General Medical Council guidance states that “all possible efforts must be made to ensure effective communication with patients.” This should involve use of independent professional interpreters:

- Consider patient preference regarding interpreter dialect, gender, and cultural background, as these may have an impact on trust and disclosure of information
- Book interpreters in advance, particularly for languages where it may be more difficult to find an interpreter
- Record the names and identification numbers of interpreters to allow rebooking and continuity.

In the consultation, enable effective communication by being compassionate, listening actively, gaining trust, and building rapport. Whenever possible, offer longer appointment times and follow-up appointments with the same healthcare professional to enable this, to ensure all health concerns are addressed, and to provide continuity of care.

When referring to specialist services, be open with patients about expected wait times to help manage expectations.

*“It is hard for me, telling my story again and again. My doctor knows what happened to me. It is better for me. I don’t have to explain everything to her every time.”*

Asylum seeker from Afghanistan.

The following section focuses on the specific needs of migrants from Afghanistan.

For migrants from Ukraine, box 3 summarises the current healthcare recommendations.

Box 3 **Clinical considerations for migrants from Ukraine**

The ECDC recommends that current healthcare provision for displaced populations from Ukraine^9^ (predominantly women, children, and older people at the time of writing) in transit and bordering countries includes access to emergency care, addressing basic needs (food, shelter, ensuring supply of medicines and medical equipment), and access to healthcare professionals. Preventing interruptions in medical supplies and care are essential to avoid excess mortality and morbidity in the coming weeks from cardiovascular disease, and chronic infectious and non-infectious diseases.^10^ In the longer term, a more holistic approach might be needed; one that considers catch-up vaccination for both children and older groups (including for polio and measles, as outbreaks of these conditions occur in Ukraine),^9^ and access to host health systems.^11^

## What clinical issues to consider?

**Fig 1:**
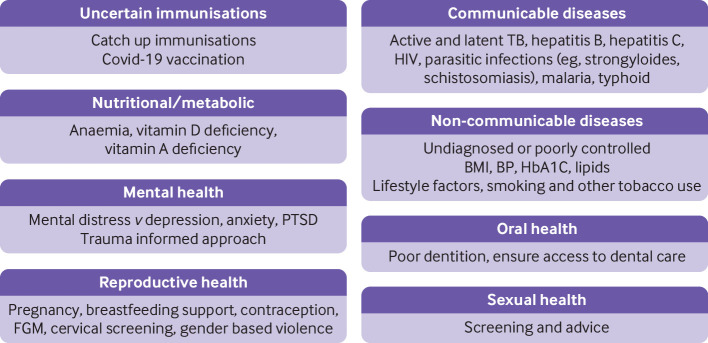
Key health considerations when assessing newly arrived migrants, refugees, and asylum seekers

Refugees are often described as facing a “triple burden” of infectious diseases, non-communicable diseases, and mental health issues. Some conditions “cluster,” owing to shared exposure to life threatening events, epidemiological burden in the country of origin, and risk factors related to the journey to the host country (examples might include diabetes, depression, and poverty, or diabetes, obesity, and lack of social network) (fig 1).^12^ Migrants’ health may deteriorate in the host country because of socioeconomic challenges, substandard accommodation, lack of digital access or digital literacy, and restricted access to healthcare, education, and labour opportunities.^13^

### Communicable disease

The UKHSA^8^ and the European Centre for Disease Prevention and Control (ECDC) offer up-to-date advice on screening for communicable diseases.^14^

The United Nations High Commissioner for Refugees and International Organisation for Migration prescribe pre-entry testing for hepatitis B and C and chest radiography for tuberculosis (TB), but this is not possible for people leaving in emergency evacuation. Furthermore, a delay often occurs between testing and travel of resettled refugees; hence repeat testing may often be required in the host country.

#### TB screening

The incidence of TB in Afghanistan is ~189 per 100 000 population.^15^ UKHSA suggests using chest radiography to screen people who are newly arrived from Afghanistan for active TB.^5^ However most cases of active TB in the UK are likely due to reactivation of latent infection (LTBI). The National Institute for Health and Care Excellence (NICE) recommends that new entrants aged under 65 from high incidence countries, such as Afghanistan, are screened for latent TB using interferon-gamma release assay via a single blood test; this is most suitable for underserved groups (including refugees and asylum seekers).^16^ In England, UKHSA’s latent TB testing and treatment programme exists in primary care for people aged 18-35, who have arrived from high incidence countries (including Afghanistan) within the last five years.^17^ Uptake of screening is limited in some migrant groups.^14^ Challenging the stigma and misconceptions around TB may help to address this.^18^ For children, contact local TB services regarding the pathways for LTBI screening.

#### Hepatitis B screening

The ECDC recommends screening for hepatitis B in migrants from countries with intermediate (HBsAg prevalence ≥2%) and high (HBsAg prevalence ≥5%) prevalence.^5^ ^14^ The prevalence of hepatitis B in Afghanistan is considered relatively high.^5^

#### Strongyloidiasis screening

Afghanistan has a high probability of being endemic for *Strongyloides*, a potentially serious but commonly asymptomatic infection. ECDC guidance recommends serological screening for strongyloidiasis,^14^ irrespective of number of years since leaving endemic countries, particularly in individuals who are immunosuppressed.

#### Hepatitis C and HIV screening

Afghanistan is not considered to have a high prevalence of HIV or to be HCV endemic, and therefore routine screening on arrival is not warranted.^14^ Consider testing if other risk factors are present.

#### Sexual health screening

When warranted, offer a full sexual health screen—including testing for HIV, hepatitis B and C, syphilis, chlamydia, and gonorrhoea—as part of an overall health assessment, taking care to be culturally sensitive (for example, some migrants may wish to discuss this only with health professionals of the same gender). Consider that if the person has a history of sexual assault or rape; they may struggle to disclose their trauma. A trauma informed approach is recommended (see below).

#### Patients who are febrile or unwell

Consider a wide range of differentials, including the infections mentioned above, as well as malaria and typhoid.

#### Catch up vaccination

Some groups may be under-immunised and/or require additional vaccines to align them with host countries’ vaccination schedules.^19^ The World Health Organization’s Immunisation Agenda 2030 calls for greater emphasis on catch up vaccination across the life course, ie, seeking every opportunity to catch up missed vaccines, doses, and boosters in children, adolescents, and older individuals. Polio is endemic in Afghanistan (41 cases reported in 2021) and measles outbreaks still occur.^20^

**Fig 2:**
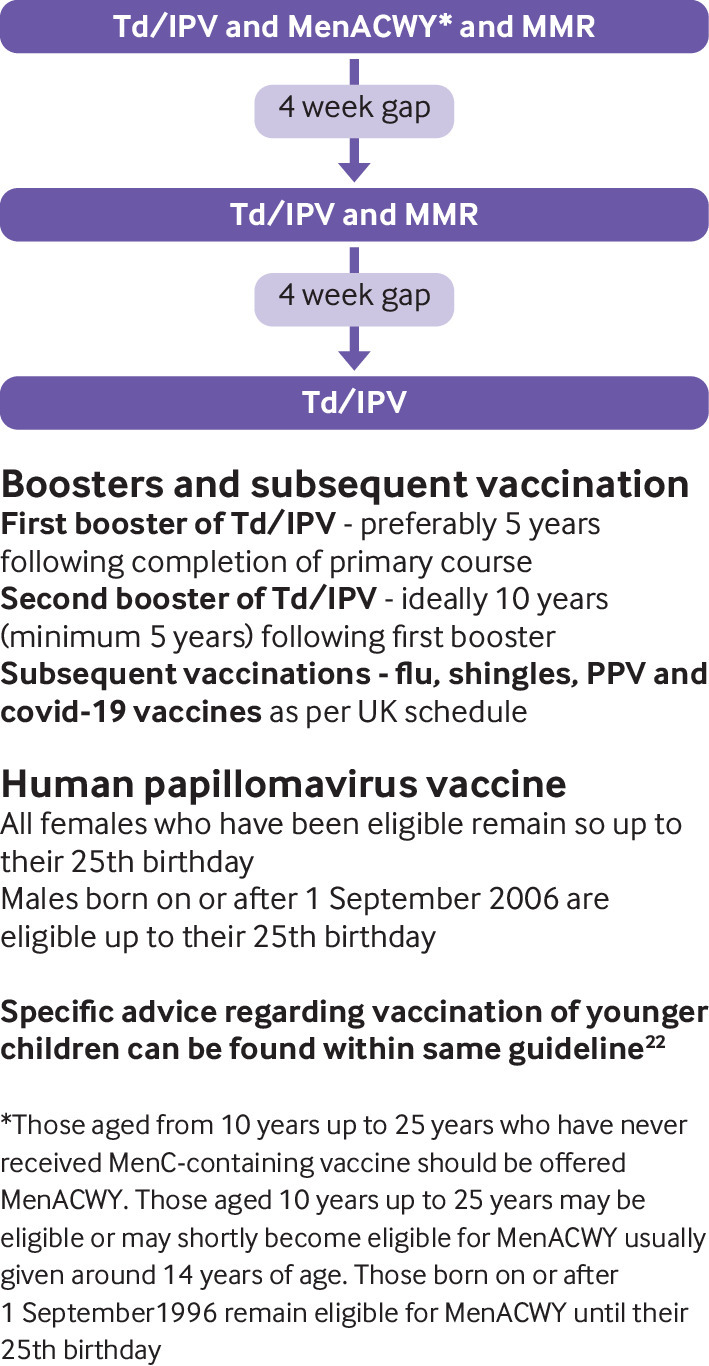
Catch up vaccinations to consider in migrants aged 10 and older. Reproduced from^22^ MMR=measles, mumps, rubella; Td/IPV=tetanus, diphtheria, polio; HPV: human papillomavirus vaccine; PPV=pneumococcal vaccine; MENACWY=meningococcal conjugate vaccine

The UKHSA advises to assume that patients are unimmunised if they are unable to provide reliable written or verbal vaccination history, and to offer vaccination according to the host country’s vaccination schedule.^21^ The UK catch up vaccination schedule for migrants aged 10 years and older is summarised in figure 2.

Some populations might have a low uptake of vaccination for covid-19 (eg, survey data using non-probability convenience sampling found nearly a third of people surveyed in Afghanistan showed limited intent to vaccinate against covid-19^23^). Consider strategies to overcome any barriers to health or vaccine systems (including vaccine hesitancy), eg, through outreach, longer appointment times, and translated patient information.^24^ A toolkit to improve vaccine uptake is available from Doctors of the World (see box, ‘Additional educational resources’) and ECDC.^25^ ^26^ The UKHSA provides guidance on managing people who may have been vaccinated in other countries.^27^

Offer written information about testing, treatment, and vaccination for covid-19 in the patient’s language.

### Non-communicable disease

Some patients may present with poorly controlled non-communicable diseases (NCDs), or may be experiencing complications owing to interruption or lack of medical care, loss of medication, and limited access to, or knowledge about, health systems in the host country.^8^ 2016 data from WHO suggest that around 8% of people in Afghanistan had diabetes; ~14% were overweight; ~2% were obese; and, in 2010, NCDs accounted for 35% of deaths.^28^ WHO also estimates that 35% of men in Afghanistan smoke.^29^ A 2021 cross sectional study of refugees from Afghanistan in Iran noted that 94% had less than adequate fruit or vegetable consumption, 20% had hypertension, 51% had central obesity, and 69% had dyslipidaemia.^30^

#### Smoking

Ask about any tobacco consumption, including sheesha (tobacco smoked through a hookah) and naswar (powdered tobacco usually placed inside cheeks).

#### Nutritional and metabolic considerations

Consider nutritional and metabolic conditions (including anaemia in preschool children and in adults) in all newly arrived migrants (look for pallor, glossitis, dry skin/hair, symptoms of anaemia, etc).^5^ If clinically indicated, request iron studies, haematinics, and haemoglobinopathy screening (to check for thalassaemia). Assess for vitamin A deficiency (dry eyes, dry skin/hair, poor night vision/other sight problems). Consider vitamin D testing and/or supplementation, especially if risk factors (such as skin pigmentation and limited sun exposure owing to cultural dress) are present. Consider calculation of body mass index, checking blood pressure, and testing for diabetes and hyperlipidaemia.^5^ In children, plot serial weight and height on growth charts, monitor for faltering growth, and consider vitamin supplementation (A, C, and D) for children aged 0-4 (in the UK, this is available for free with the “Healthy Start” programme).^31^ Offer lifestyle advice with careful contextualisation and awareness of social, financial, and practical constraints.

#### Oral health

Oral health is often overlooked and some migrant groups may not have had access to dentists for a considerable time. Explore dental symptoms and encourage attendance for routine dental care at the earliest opportunity.^8^ All forms of tobacco consumption (see above) can affect oral health.

#### Medication history

Ask about any pre-existing treatment. Patients may be taking medication that is not available in the host country or has an unrecognisable name. In these cases, switch according to best practice guidelines and review response to treatment. Explain where and how to collect medication, how to order repeat prescriptions, and advise about any prescription charges and exemptions.

### Mental health

Mental distress does not always equate to mental illness. Cultural and linguistic differences can inappropriately increase the likelihood of a diagnosis of mental illness.^32^ On top of the multiple challenges of adapting to living in a new country, people may have experienced conflict, violence, multiple losses, torture, sexual assault, and/or be at risk of exploitative situations.

Be mindful of cultural perceptions/stigma and impacts of mental health presentations and diagnoses.^33^

Patients may present with somatic symptoms, such as headaches, chest pain, back pain, and abdominal symptoms.

#### Use a trauma informed approach

**Fig 3:**
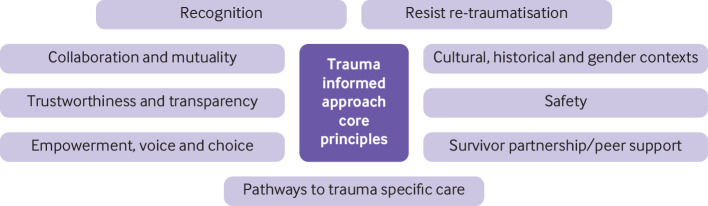
The core principles of trauma informed approaches, which acknowledge the impact previous trauma may have had on an individual, and offer care in the context of this. The focus is on developing relationships, understanding experiences, and building resilience to provide a strong foundation for recovery^34^

Ask open questions while remaining sensitive to patient cues about topics they may not want to discuss (full details are not essential to assess mental health adequately). Use a trauma informed approach (fig 3). Be alert to symptoms of depression and anxiety, and in the context of trauma, specifically inquire about symptoms of post-traumatic stress disorder.^35^

#### Ask about trauma

Do not be afraid to ask about trauma, but be respectful of potential re-traumatisation. Advise patients that you will be asking difficult questions that they can choose not to answer. Move at the patient’s pace, listen, and ensure post-disclosure support.^34^

### Reproductive health

When appropriate, consider pregnancy and refer to antenatal services, offer vitamins (folic acid, vitamins C and D), and offer breastfeeding support; contraception, and explain and offer cervical screening.

### Abusive situations

People may be victims of gender based violence, domestic violence (spousal or interfamily violence), honour based violence, trafficking or forced migration, modern slavery, and forced marriages.^36^ Follow relevant local safeguarding procedures and seek advice if you have concerns.

### Female genital mutilation

Consider female genital mutilation (FGM), particularly if the patient is from a region where it is known to be practised, if they have a family history of FGM, or if they present with genitourinary symptoms. Limited information is available about the practice of FGM in Afghanistan. Further resources are available through the UK Home Office’s FGM unit.^37^

## How can we improve access to care and services?

Use a holistic and patient centred approach to assess patients’ needs. Initiatives including the Doctors of the World UK’s Safe Surgeries’ Toolkit offer advice on overcoming common barriers. Other suggestions include:

- Better support for GPs in understanding the diverse health needs in different migrant groups
- Digital tools that offer GPs immediate tailored advice on screening and catch up vaccines, based on country of origin (currently being explored^6^ ^38^)
- Record patient’s spoken and written language preferences, avoiding assumptions regarding literacy
- Encourage staff familiarity with translated written resources (see box, “Patient resources”)
- Training and understanding of best practice in use of professional interpreters^5^
- Offer social prescribing to enable access to relevant services and community groups, such as immigration related support, language classes, financial and educational support, and community participation and socialisation opportunities^39^
- Due to challenging financial situations, signpost to voluntary services and food banks, and explain entitlements to welfare benefits, support with prescription fees, or transport to appointments
- Link newly arrived people and individual family members to the local community (eg, host country language classes, acculturation opportunities).^8^

**Patient perspectives**

“My wife, son, and I left Afghanistan during the evacuation following the Taliban takeover of Kabul. It was a traumatic experience in many ways—there was not enough time to plan, we had to leave all our essential personal belongings behind, and had to go through Taliban lines. I was injured with a gun barrel by a Taliban member, and my wife and son were terrified. We were relieved after arriving in the UK. We were given a warm welcome and the feeling of being fully taken care of, and we stayed in a quarantine hotel for 10 days. We worried about those we had left behind, feeling sad for our country, for losing everything we had worked very hard for—our home, job, possessions, family, and friends—perhaps the confinement in the hotel made our feelings worse. After this, we were moved to a bridging hotel. Health services were not automatic. I am fluent in English and so I was able to proactively seek care for my pregnant wife, but others may not have done. By the time the date of her scan arrived, we had moved again to our permanent address. It would be useful to have all the records linked up to save repeating our story and blood tests, and to have some written information in our own language. We have now had a scan and are pleased our baby is fine. We are grateful that we are together and safe.”–*Anonymous*

“Immigration is a difficult choice. There is a complete break from family, traditions, and customs, and a transition into a new life. I was a sick single mother with four children and took refuge in a new country after a long journey of suffering in my country. During this time, we suffered physical and psychological problems. Everything was different—language, community, country. Even my fears were different. The first impression with healthcare can change a lot in the new life of a refugee. A smile and kindness can open many doors towards a future and close the door of fear and hesitation. My doctors were able to gain the trust of my children, breaking the barrier of fear from a doctor, because a doctor in my country means injection. I could speak a little English but being able to speak in my own language with an interpreter and seeing the same doctor helped me a lot. Now my daughters are studying science and want to be doctors, too. I am studying in college and will publish my online newspaper soon.”–*Haja Ahmed*

**Additional educational resources**

**(All freely available and do not require registration)**

BMA Guidance: Refugee and asylum patient health toolkit: https://www.bma.org.uk/advice-and-support/ethics/refugees-overseas-visitors-and-vulnerable-migrants/refugee-and-asylum-seeker-patient-health-toolkit https://www.bma.org.uk/advice-and-support/ethics/refugees-overseas-visitors-and-vulnerable-migrants/refugee-and-asylum-seeker-patient-health-toolkit
Liverpool John Moores University resources: https://www.ljmu.ac.uk/microsites/resources-for-professionals-who-support-asylum-seekers-and-refugees
Public Health England, Afghanistan, Migrant Health Guide: https://www.gov.uk/guidance/afghanistan-migrant-health-guide
Doctors of the World, Safe Surgeries Toolkit for general practices to provide a welcoming and equitable service for all patients and address the barriers faced by migrants in vulnerable circumstances: https://www.doctorsoftheworld.org.uk/what-we-stand-for/supporting-medics/safe-surgeries-initiative/safe-surgeries-toolkit/

**Patient resources**

**(All are freely available and do not require registration)**

**Translated health information**

- Doctors of the World: patient information leaflets in multiple languages. Resources include how to register with a GP, migrants’ right to healthcare, covid-19 vaccination, wellbeing, self-care, and keeping young people healthy: https://www.doctorsoftheworld.org.uk/translated-health-information/
- Doctors of the World: resources specifically for Afghan patients are available in Dari, Pashto, Farsi, or Urdu: https://www.doctorsoftheworld.org.uk/news/list-of-multilingual-resources-for-afghans/
- Refugee Council: patient communication cards, introduction cards, guide to using the GP, and which NHS service to use in multiple languages, including Dari: https://www.refugeecouncil.org.uk/get-support/services/therapeutic-wellbeing-resources/

**Family tracing**

- Red Cross International Family Tracing helps find missing relatives abroad who have been separated by war, natural disaster, or migration: https://www.redcross.org.uk/get-help/find-missing-family. UK Freephone telephone number +44 808 196 3651

**General advice, support, and counselling**

- Refugee Council offers advice, counselling, and practical support to refugees and asylum seekers: https://refugeecouncil.org.uk/
- Refugee Council InfoLine signposts refugees and asylum seekers to relevant organisations: UK Freephone telephone number +44 808 196 7272, Monday-Thursday 9 30 am to 12 30 pm
- Barnardos Boloh Helpline offers advice, signposting, and emotional support to asylum seekers. UK freephone telephone number +44 800 151 2605. Monday-Friday 10 am to 8 pm, Saturday 10 am to 3 pm. Boloh.helpline@barnardos.org.uk

**Support and information specifically for people from Afghanistan**

- https://refugeecouncil.org.uk/information/information-for-afghans/guidance-and-support/support-and-information-for-people-affected-by-the-crisis-in-afghanistan/
- UK government welcome pack for Afghan locally employed staff: https://assets.publishing.service.gov.uk/government/uploads/system/uploads/attachment_data/file/1004550/Afghanistan_LES_Welcome_Pack.pdf

**Support for people who have experienced torture**

- Freedom from Torture provides therapy and support for people who have experienced torture: https://www.freedomfromtorture.org/help-for-survivors. UK telephone number +44 207 697 7777
- Helen Bamber Foundation provides therapy and support for people who have experienced torture and/or been trafficked, and other forms of extreme physical, sexual, and psychological violence: https://www.helenbamber.org/. UK telephone number +44 203 058 2020. reception@helenbamber.org

**Help with prescription charges, dental treatment, and sight tests**

- Refugees, asylum seekers, and other migrants can apply for the NHS Low Income scheme, which helps pay for some medical costs, including prescription charges, dental treatment, and sight tests: HC1 online application form: https://services.nhsbsa.nhs.uk/apply-for-help-with-nhs-costs/apply-online . Advice is available is different languages on the telephone or online. UK freephone telephone number +300 330 13 43. https://www.nhsbsa.nhs.uk/advice-other-languages

**Education into practice**

- What changes could your department make to ensure migrants’ needs are met? For example, what would a “welcome pack” from your practice look like? What cultural competence training do your staff members require?
- What interpreting services and translated health information do your clinical and administrative staff know how to access and use effectively?
- How would you explore the experiences and journey that a refugee patient has taken?

**How patients were involved in the creation of this article**

One of the authors, Haja Ahmed, a journalist from Sudan, is a refugee who resettled in the UK with her four children on a government resettlement scheme five years ago. She provided her personal insights and experiences of arriving in a new country and navigating her way through the NHS.

We also spoke to several refugees and asylum seekers from Afghanistan, specifically asking what issues they had faced with accessing healthcare, and what would be helpful for clinicians to know when seeing newly arrived refugees from Afghanistan. Their advice led to several amendments, including highlighting how healthcare workers’ interaction substantially influences patients’ new lives in a new country, and the anonymous quotes included.

Comments from external patient reviews echoed the views of other patient contributors and were also incorporated.

**How this article was created**

We combined advice from relevant guidelines and advice documents from Public Health England (PHE, the now UK Health Security Agency), NICE, The European Centre for Disease Prevention and Control, and the World Health Organization’s Regional Office for Europe, with our professional and clinical experience. We also searched PubMed and the Cochrane Database of Systematic reviews for articles published in English. We used the search terms “Afghan,” “Afghanistan,” “Refugee,” “Asylum Seeker,” and “Migrant” in combination with the terms “Health” or “Healthcare.” We limited the search to peer reviewed systematic reviews published over the past 10 years to capture recently available evidence and guidelines. We found 298 articles to inform this article creation.

## Notes

We thank Angela Burnett (Freedom from Torture and GP), Dominik Zenner (Queen Mary’s University of London and GP), and Anna Miller (Doctors of the World UK) for their expert insights and advice regarding specific sections of this article. We thank the Health Inclusion team at Guy’s and St Thomas’ NHS Trust for its support and sharing of front line clinical expertise, particularly Krishna Misra, joint Clinical Lead of Refugee and Asylum Seeker Services (GP). SH acknowledges funding from the National Institute for Health Research (NIHR Advanced Fellowship NIHR300072), the Academy of Medical Sciences (SBF005\1111), the Novo Nordisk Foundation (Mobility—Global Medicine and Health Research grant), and the World Health Organization. FK is supported by a Health Education England/National Institute for Health Research (NIHR) academic clinical fellowship.

Contributorship and guarantor: FK, SH, and SM conceived the article and are guarantors. SM was the contact for patient involvement. FK and SM contributed equally to this paper and are joint first authors. All authors made substantial contributions to the text and were involved in drafting and revising the work and in final approval. All authors accept responsibility for the accuracy and integrity of the work.

Provenance and peer review: commissioned, based on an idea from the author; externally peer reviewed
